# An Uncommon Presentation of Metastatic Melanoma: A Case Report

**DOI:** 10.1097/MD.0000000000000319

**Published:** 2015-02-20

**Authors:** Isabella Reccia, Adolfo Pisanu, Mauro Podda, Alessandro Uccheddu

**Affiliations:** From the Department of Surgery, Clinica Chirurgica, University of Cagliari, Policlinico Universitario di Monserrato, Sestu (CA), Italy (IR, AP, MP, AU).

## Abstract

Metastases to the spleen are rare and are generally part of a multi-visceral metastatic disease. The most common sources of splenic metastases include breast, lung and colorectal malignancies as well as melanoma and ovarian carcinoma. Solitary splenic metastasis is very uncommon.

We present a case of a 44-year-old man who presented at our department for gallstones symptoms. He had a past medical history of neck cutaneous melanoma (T3bN0M0—Stage IIb). He had not attended follow-up schedule for personal reasons. However, abdominal ultrasound revealed the presence of a solitary solid lesion in the spleen. Preoperative workup was completed with CT scan that confirmed the presence of a large splenic lesion with subcapsular fluid collection, also compatible with a post-traumatic lesion.

Preoperative findings could not exclude malignancy and patient was therefore submitted to surgery. At laparoscopy, a condition of peritoneal melanosis was present. Splenectomy was carried out. Histological report confirmed the peritoneal melanosis and the diagnosis of metastatic spleen lesion from melanoma. Patient was observed, but died of metastatic disease 14 months after surgery.

Splenic metastases are uncommon. Isolated metastases from melanoma are rare and could be found several months after primary diagnosis of melanoma. Surgery remains the most effective treatment, especially for metachronous disease, offering the best chance of long-term survival. Prognosis remains poor, as metachronous disease is indicative of aggressive widespread of the disease.

## INTRODUCTION

Metastases to the spleen are rare and are generally part of a multi-visceral metastatic disease. The most common sources of splenic metastases include breast, lung, and colorectal malignancies as well as melanoma and ovarian carcinoma.^1,2^ Solitary splenic metastasis is very uncommon.^3,4^ We present a case of a giant solitary splenic metastasis from melanoma associated with peritoneal melanosis.

## CASE PRESENTATION

**FIGURE 1:**
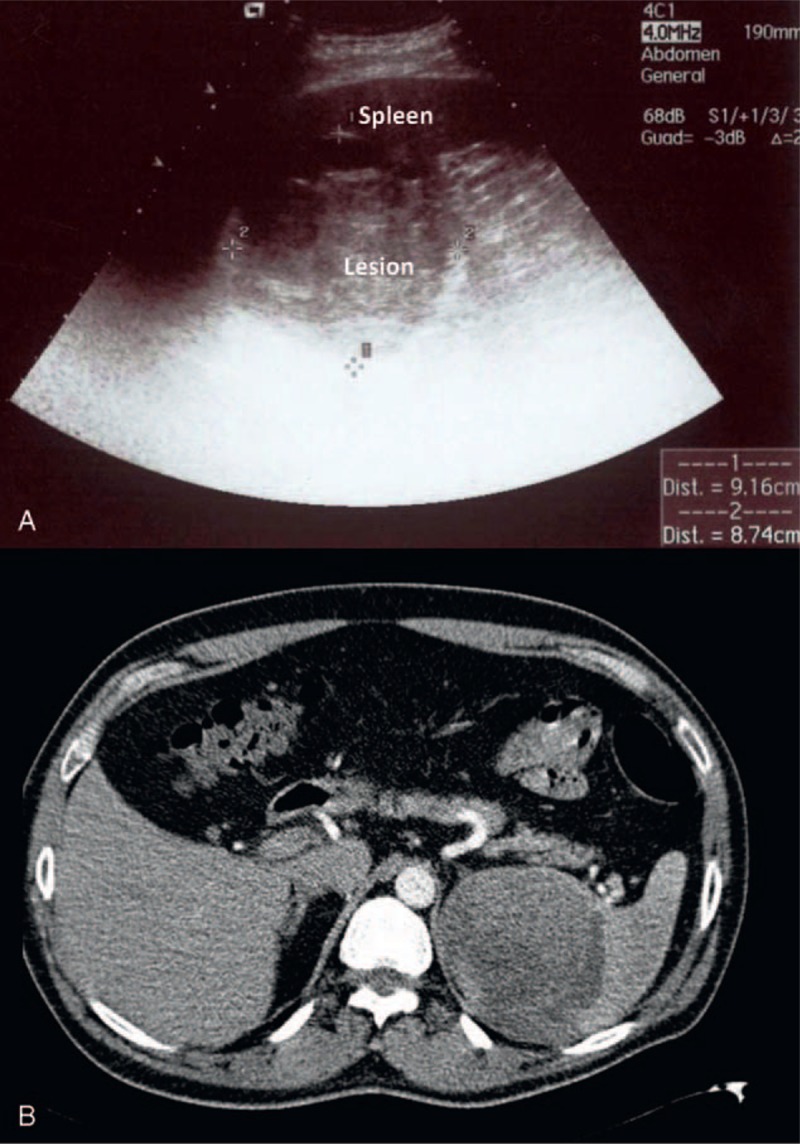
(A) Ultrasound of the spleen showing a large 9 × 8 cm solid lesion at the upper pole, with mixed echogenicity, prevalently hyperechoic, and almost no vascularization; (B) Arterial phase helical CT scan showing a 76 × 76 mm round mass, with dishomogeneous hypodense and high-density areas located at the upper pole of the spleen, with a thick subcapsular fluid collection at the inferior pole (not visible in this section).

**FIGURE 2:**
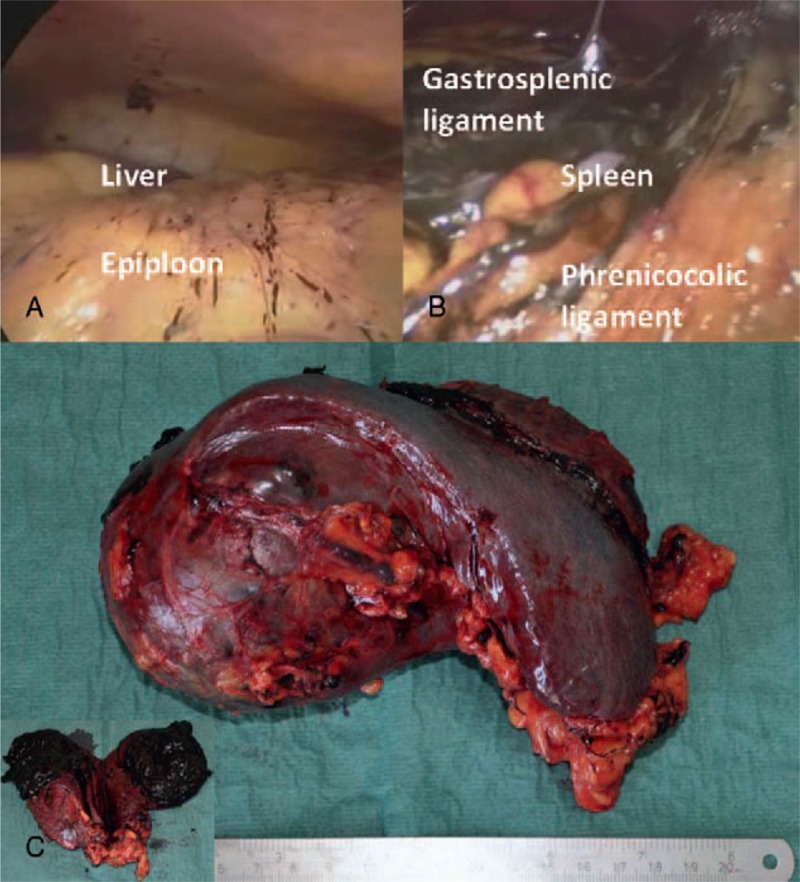
(A) Intraoperative findings of focal peritoneal melanosis on the epiploon and on the peritoneal surface of the right diaphragmatic peritoneum; (B) massive amount of peritoneal melanosis around the spleen, on gastrosplenic and phrenicocolic ligaments; (C) Surgical specimen showing a large brownish irregular round mass that protrudes above the surface at the upper pole of the spleen; a cut surface shows the dishomogeneous appearance and the intense dark brown color of the lesion, with soft solid component and areas of colliquation.

A 44-year-old Caucasian man presented to our surgical department after 1 week of epigastric and right upper abdominal pain radiating to the right shoulder. Family history was non-contributory. He had no associated comorbidities. Other complains included bloating and nausea. He had a past medical history of neck cutaneous melanoma (2 years before), with sentinel lymph node biopsy negative (T3bN0M0—Stage IIb), but he did not attend follow-up schedule for personal reasons. Physical examination revealed mild tenderness in the right upper abdominal quadrant. No organomegaly or masses were clinically identified. Laboratory tests showed only a mild leukocytosis and moderate level of lactate dehydrogenase—LDH (488 UI/L, range 208–378). Gallstones were suspected in the view of patient's symptoms. However, admission abdominal ultrasound (US) of the abdomen revealed the presence of a round solid mass at the upper pole of the spleen (9 × 8 cm), with mixed echogenicity and almost no vascularization (Figure 1a). Cholelithiasis was also confirmed by abdominal US. The patient was therefore submitted to Computer Tomography (CT) scan, which showed a round mass (76 × 76 mm) at the upper pole of the spleen, which was dishomogeneous with hypodense and high-density areas, and with a subcapsular fluid collection (Figure 1b). CT scan appearance could also be compatible with a splenic hematoma, but the patient denied any history of trauma and had a past medical history of cancer. Chest X-ray was normal. Because of the US and CT unclear findings and the patient's history, he was referred for surgery for exploratory laparoscopy, cholecystectomy, and splenectomy. The procedure was first attempted laparoscopically with a full lateral right decubitus position, as previously described elsewhere for laparoscopic splenectomy.^5^ However, on surgical exploration, a diffuse, brown peritoneal pigmentation was found (Figure 2a–b). At frozen section lesions were composed of clusters of pigment-laden macrophages within the peritoneum, compatible with a condition of peritoneal melanosis. No cancer cells were identified. The splenic lesion had a brownish color with bulging into the splenic capsule at the upper pole, without macroscopic evidence of capsular breach. Because the appearance at exploration, the strong suspicion of malignancy, the presence of extensive adhesions and the risk of lesion rupture the procedure was converted to an open approach. Postoperative recovery was uneventful. Pathologic examination showed a parenchymal diffusely necrotic mass rich in melanotic pigment (Figure 2c), with focal capsular invasion and satellite nodules of strongly atypical neoplastic cells (Melan A+, HMB-45+, and S100+). The definitive diagnosis of splenic metastasis from melanoma and peritoneal melanosis was thus made. The patient was evaluated by the oncologists and submitted to brain magnetic resonance imaging (MRI) and positron emission tomography (PET)/CT, which were both negative for metastatic disease. Patient had no evidence of residual disease and was observed. However, at 1 year from surgery patient developed new neurologic symptoms and brain MRI was performed, showing the presence of multiple brain metastases. The patient rapidly developed disseminated disease and died of the disease at 14 months from splenectomy and at 38 months from initial diagnosis of melanoma.

## DISCUSSION

Splenic tumors are uncommon and present a diagnostic dilemma. Splenic metastases are relatively rare and can be found even 2 to 3 years after detection of primary tumor.^1,3,4^ Rarity might be explained by mechanical factors impeding hematogenous implantation of neoplastic cells (angled course of the splenic artery, blood flow, splenic capsule, lack in afferent lymphatics, and contractile properties of the spleen).^1,3^ Splenic metastases are part of multi-visceral metastatic disease, but are often asymptomatic and incidentally found.^1^ Isolated metastases are rare.^3^ Diagnosis should always be suspected when past medical history is positive for cancer. Imaging characteristics include the presence of a solitary spleen lesion, usually hypoechoic at sonography and hypodense at CT, with inhomogeneous contrast-enhancement as for the presence of necrosis.^1^ Contrast-enhanced CT is the imaging modality of choice for the evaluation of several abdominal conditions. For focal splenic lesions, contrast-enhanced CT has a high diagnostic accuracy for detection and characterization of these lesions.^6^ However, a multimodality approach is often needed to combine the key features of different imaging modalities.^7^ For example, MRI can be helpful in differentiating benign or malignant lesions due to the signal-intensity and the depiction of internal hemorrhage.^8^ Finally, contrast-enhanced US can be used to differentiate benign from malignant solitary splenic lesions, but differentiation between lymphoma and metastases is almost impossible, even if necrosis is typical of metastatic disease.^9^

Despite that, biopsy may be necessary for definitive diagnosis when imaging findings are nonspecific. Fine needle aspiration (FNA) has been advocated as a safe and useful diagnostic modality that allows a cytological diagnosis of splenic lesions and helps in defining the most appropriate treatment, especially in cancer patients.^10,11^ In the case of metastatic melanoma, the suspicion of distant metastasis should be confirmed by FNA cytology, core or open biopsy, except for brain metastases that are usually treated without routine biopsy.^12^ In our case, the presence of a subcapsular fluid collection indicated a partial laceration of the lesion and FNA was thus not performed due to the high risk of open rupture and possible metastatic dissemination. Moreover, patient had no clinical evidence of systemic disease at the time and resection of solitary visceral metastasis from melanoma, if feasible, is an accepted procedure. However, since isolated melanoma metastases are rare and could represent the first sign of systemic recurrence, a short period of observation or systemic treatment can be done to exclude the presence of a multi-site metastatic disease.^12^ Biopsy can thus allow a genetic analysis especially when adjuvant therapy, targeted therapy or enrollment in clinical trial is considered.

Treatment options for patients with stage IV melanoma include immunotherapy, targeted therapy, chemotherapy, biochemotherapy, radiation therapy, surgery for isolated and limited metastases, and participation in clinical trials. Due to advances in understanding of melanoma biology, immunotherapy and target therapy, alone or in combination, have shown promising results in the treatment of advanced melanoma.^13^ Selection of most appropriate treatment depends on multiple factors, including patient's performance status and preferences, site (ie, brain) and extension of metastases, and the presence of specific genetic mutations (BRAF, n-RAS, and c-KIT).

Surgery remains the most effective treatment for malignant melanoma, especially in patients with hematogenous dissemination of disease, when it is resectable.^12,14^ Complete surgical resection in patients with solitary metastasis appears to offer the best chance of long-term survival.^14^ A complete metastatic workup is mandatory, including complete evaluation of specific signs and symptoms when present.^12^ Evaluation of patients with metastatic melanoma should always rely on the combination of appropriate clinical information and accurate workup that has to include chest and abdominal-pelvic CT scan, FNA or biopsy, brain MRI in the presence of neurologic symptoms, clinical suspicion of brain metastases or if results could influence decision, and serum LDH.^12^ In more advanced stages of the disease, stages III and IV, PET/CT scan can be useful to detect occult metastatic disease or to better characterize uncertain findings at CT scan.^15^ It has been also demonstrated that PET/CT scan can impact the decision making process and the decision made in patients with advanced melanoma.^16,17^ PET/CT scan can be suggested prior to surgery in apparently resectable patients at high risk of disseminated disease.^16^

For metastatic resectable disease, adjuvant therapy is not routinely recommended after surgery, but it can be offered to patients on clinical trials.^12^ Treatment with high doses of interferon alpha (INFα) has been proposed as adjuvant therapy in patients with stages II to III resected melanoma with potential benefits on survival.^18–20^ For resected metastatic melanoma (stage IV), patients should be treated with INFα only inside clinical trials.^12^

Peritoneal melanosis is a rare pigmented condition of the peritoneum, which is visible to the naked eyes, frequently associated with ovarian cystic teratomas.^21^ Association with melanoma is rarer.^21^ Melanoma has a high malignant potential and can spread almost everywhere. Cutaneous melanoma is more aggressive, with a relatively worse prognosis for a high recurrence rate and higher mortality.^22^ Skin melanoma has the highest rate of splenic metastases per primary tumor. Metachronous disease has a more favorable prognosis. However, it is indicative of an aggressive widespread disease with a poor prognosis.^2^ LDH level is a valuable indicator of disease recurrence and must be part of the assessment for advanced due to its important prognostic role.^12,23^

In conclusion, peritoneal melanosis is a benign condition and association with melanoma is unusual. Splenic metastases are uncommon. Isolated metastases from melanoma are rare and could be found several months after primary diagnosis of melanoma. An isolated solid splenic lesion in a patient with a past history of malignancy is highly suspicious for metastatic disease. Surgery remains the most effective treatment, especially for metachronous disease, offering the best chance of long-term survival.
